# Pelvic radiography as a non-invasive screening tool for hiatal hernia in children with cerebral palsy

**DOI:** 10.1097/MD.0000000000029522

**Published:** 2022-08-19

**Authors:** In Hyuk Yoo, Hye Ran Yang

**Affiliations:** a Department of Pediatrics, Seoul St. Mary's Hospital, School of Medicine, The Catholic University of Korea, Seoul, Korea; b Department of Pediatrics, Seoul National University Bundang Hospital, Seongnam, Korea; c Department of Pediatrics, Seoul National University College of Medicine, Seoul, Korea.

**Keywords:** cerebral palsy, child, gastroesophageal reflux, hernia, hiatal, hip dislocation

## Abstract

The diagnosis of hiatal hernia (HH), causing severe gastroesophageal reflux disease and complications in children with cerebral palsy (CP) is cumbersome because invasive investigations are required for diagnosis. Hip displacement, one of the most common complications in children with CP, can be diagnosed with a simple pelvic radiograph. This study aimed to evaluate the association between the severity of hip displacement and HH and the diagnostic accuracy of Reimers’ hip migration percentage (MP) on pelvic radiography in assessing the presence of HH. A total of 52 children with CP (27 boys, 25 girls; mean age, 6.3 years; range, 0.6–17.4 years) who underwent esophagogastroduodenoscopy, upper gastrointestinal series and pelvic radiography between March 2013 and February 2020 were recruited. Demographic and clinical characteristics, as well as endoscopic and radiological findings, were evaluated and statistically analyzed. HH was defined as ≥ 2 cm proximal displacement of the gastroesophageal junction identified in esophagogastroduodenoscopy or upper gastrointestinal series, and MP was calculated by evaluating the pelvic radiograph. Of the 52 children enrolled in this study, HH was diagnosed in 18 children (34.6%). When the patients were classified and analyzed according to the MP result, HH was observed in 10%, 26.7%, and 70.6% in MP <33%, MP 33%–39%, and MP > 40% groups, respectively (*P* < .001). The optimal MP cutoff of 36.5% distinguished pediatric CP patients with HH from those without HH with a sensitivity of 78%, specificity of 68%, a positive predictive value of 56.0%, and a negative predictive value of 85.2%, respectively. The application of MP and the severity of hip displacement, which can be easily measured by simple radiography, may be useful and reliable in screening for detecting HH in children with CP. Retrospectively registered. This study was approved by the Institutional Review Board of Seoul National University Bundang Hospital (IRB No. B-2007-627-106).

## 1. Introduction

Gastroesophageal reflux disease (GERD) is common in children with cerebral palsy (CP) and, if severe, can have a significant impact on their health outcomes.^[1,2]^ Aspiration pneumonia, a complication of GERD, is a leading cause of death and hospitalization in these patients, and other complications such as malnutrition and reflux esophagitis can also have a great influence on their quality of life and long term prognosis.^[3–5]^ The causes of severe GERD are complex and varied, but hiatal hernia (HH) is recently considered an important pathogenetic contributor.^[6]^

HH is defined as an axial displacement of the proximal stomach through the diaphragmatic hiatus of the diaphragm.^[6]^ HH occurs as the phrenoesophageal membrane elasticity gradually decreases over a long period of time; additionally, the repeated stress of swallowing, gastroesophageal reflux, vomiting, retching, and straining are known as aggravating factors.^[7–9]^ Thus, HH is common among older adults and is generally rare in children; however, it can occur in children with chronic conditions, such as CP or in those with a history of surgery or chemotherapy.^[10]^ HH is clinically important because HH can cause severe GERD and complications.^[11]^ The pressure barrier of the lower esophageal sphincter plays a key role in the defense mechanism against gastroesophageal reflux.^[12]^ When HH occurs, this protective mechanism weakens and the gastroesophageal reflux becomes severe, resulting in symptoms and complications of GERD such as reflux esophagitis, nutritional deficiencies, aspiration pneumonia and faltered growth.^[13–16]^ A clear association between HH and GERD has already been confirmed in pediatric studies as well as in adult studies.^[17,18]^

The reason why HH is more problematic in pediatric patients with CP is that these patients are not only a high-risk group for developing HH, but complications that may occur due to HH such as aspiration pneumonia and malnutrition have a great influence on the prognosis of these patients.^[3–5]^ Since HH usually worsens as it progresses, it is ideal to diagnose early before developing severe complications; however, it is not easy in practice because the diagnosis of HH requires invasive procedures, such as esophagogastroduodenoscopy (EGD) or upper gastrointestinal series (UGIS). Additionally, it is more difficult to suspect the presence of HH because CP patients have limited communication skills and are not able to complain of clinical symptoms that may be indicative of GERD.^[19]^ However, it is practically difficult to implement invasive diagnostic methods such as EGD and UGIS on all children with CP without any symptoms.

Hip displacement is one of the most common complications in children with CP.^[20,21]^ The cause of hip displacement in patients with CP is not completely clear. However, it is generally more common in bed-ridden patients, and factors that consistently give the wrong direction of force to the hip joint, such as spasticity, pain, and straining, are thought to be the main cause of hip displacement in patients with CP.^[22]^ Additionally, these factors are also the causative and aggravating factors of developing HH in patients with CP.^[6]^

Since hip displacement and HH have common causative and aggravating factors, it is assumed that there might be a correlation between the two diseases. If the relationship between the severity of hip displacement and the occurrence of HH could be confirmed, a simple pelvic radiograph may be used as a beneficial screening tool to identify high-risk groups of HH in pediatric patients with CP.^[23]^

Therefore, the aim of this study was to determine the association between the severity of hip displacement and the occurrence of hiatal hernia, and to evaluate the diagnostic accuracy and optimal cutoff of Reimers’ migration percentage (MP) on pelvic radiography in assessing the presence of HH.

## 2. Methods

### 2.1. Subjects

Children with CP aged ≤ 18 years, who underwent EGD, UGIS, and pelvic radiography at the Seoul National University Bundang Hospital between March 2013 and February 2020 were recruited. The patients were diagnosed with CP by pediatric neurologists; additionally, all of the study participants were bed-ridden (GMFCS Level V). The subjects were tested for gastrointestinal symptoms, such as vomiting and abdominal pain, or clinical symptoms and complications suspicious of severe GERD, such as recurrent aspiration pneumonia. HH was diagnosed by combining the results of EGD and UGIS, and the severity of the hip displacement was evaluated by calculating the Reimers’ MP on pelvic radiography. Among the study subjects, the patients under 1-year of age whose femoral head was not suitable for measuring MP were excluded, and those who had already undergone surgery for hip dislocation or HH were also excluded from the study. Finally, a total of 52 children with CP were recruited; the demographic and clinical characteristics as well as endoscopic and radiological findings were retrospectively analyzed in all subjects.

This study was approved by the Institutional Review Board of Seoul National University Bundang Hospital (IRB No. B-2007-627-106). Informed consent was waived by Institutional Review Board of Seoul National University Bundang Hospital because of the retrospective nature of the study and the analysis used anonymous clinical data.

### 2.2. Diagnosis of hiatal hernia

EGD was performed by pediatric gastroenterologists using a GIF-Q260 or GIF-XP260 scope (Olympus, Tokyo, Japan). Subsequently, the EGD images and reports were reviewed retrospectively by a pediatric gastroenterologist. The distance between the gastroesophageal junction (GEJ) and the diaphragmatic indentation was measured at the end of the EGD after deflating the stomach. The gastroesophageal flap valve grades 1–4 based on Hill classification were evaluated on the retroflexed views of EGD. HH was defined when the proximal displacement of the GEJ was definitely ≥ 2 cm and/or gastroesophageal flap valve grade 4 by Hill classification observed on a retroflexed view of EGD.^[24,25]^ Among children who did not fulfill the criteria for HH, ≥ 0.5 cm proximal displacement of the GEJ identified on EGD was defined as a short segment hiatal hernia (SSHH).

UGIS was performed in all study subjects, and the results of the UGIS were evaluated by pediatric radiologists. A predetermined quantity of barium was administered using a cup or straw, and if not possible, a feeding bottle or an enteric tube was used for testing. The esophagus and GEJ were observed during swallowing, and HH was diagnosed if the B ring or the upper part of the gastric folds reached ≥ 2 cm above the diaphragmatic indentation during quiet respiration.^[24]^

### 2.3. Measurement of Reimers’ MP

**Figure 1.**
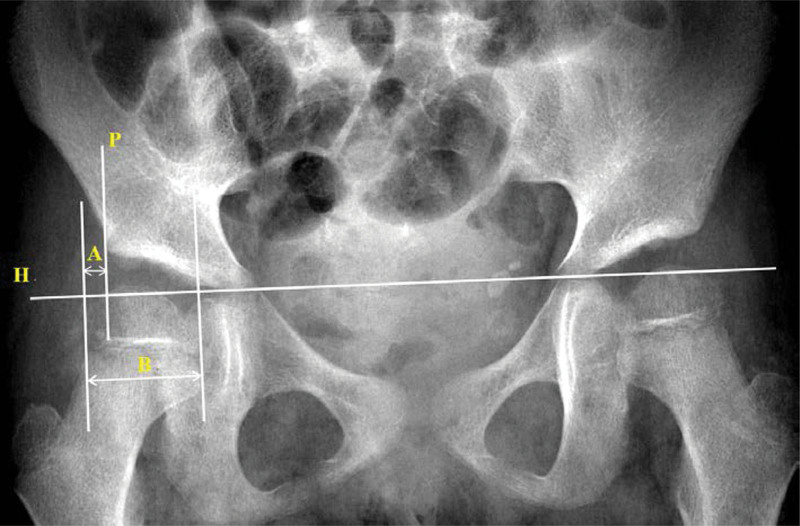
Reimers’ migration percentage values (MP). The Hilgenreiner's line (H) is the horizontal line between the triradiated cartilage of both hips. The Perkins’ line (P) is drawn perpendicular to the Hilgenreiner's line at the lateral margin of the acetabulum. The A line is without the acetabulum part of the femoral head. B line is the total diameter of the femoral head. Note that MP = A/B × 100%. MP = migration percentage.

Reimers’ MP was used to assess the lateral displacement of the hip.^[23]^ Pelvic radiographs were taken during the same hospital stay with EGD and UGIS. Additionally, the measurements were obtained by one pediatric gastroenterologist. To evaluate the MP, the Hilgenreiner's line, which is a horizontal line between the superior aspect of the triradiate cartilage, was determined. A second line, known as the Perkins’ line, was drawn perpendicular to the Hilgenreiner's line at the lateral edge of the acetabulum. Based on Perkin's line, the MP was obtained through the ratio of the lateral side of the femoral head (Fig. 1).^[21,26,27]^

According to the severity of Reimers’ MP for hip displacement, the study subjects were classified into three stages, and the clinical features and the status of HH were analyzed and compared among the three stages: MP < 33%, MP between 33% and 40%, and MP > 40%.^[28,29]^

### 2.4. Statistical analysis

All statistical analyses were performed using PASW Statistics (version 25, SPSS, Chicago, IL, USA). The Kruskal-Wallis test and linear by linear association were used to analyze the quantitative variables. The Mann-Whitney and Chi-square tests were used to analyze the differences between the no-HH and HH groups. A receiver operating characteristics plot analysis was used to evaluate the optimal MP cutoff levels. For all statistical analyses, a two-sided *P* value < .05 was considered statistically significant.

## 3. Results

### 3.1. Comparison of patient characteristics and clinical features according to the MP value

A total of 52 children (27 boys, 25 girls; mean age, 6.3 years; range, 0.6–17.4 years) were finally recruited, and 18 (34.6%) of 52 children had HH. There were no side effects related to examinations in all patients.

According to the severity of MP for hip displacement, the subjects were classified into three stages: MP < 33% (n = 20), MP between 33% and 40% (n = 15), and MP > 40% (n = 17). When comparing the patients’ age, sex, nutritional status, and presence of HH among the three groups according to the severity of MP, the patients with higher MP revealed a higher proportion of HH in children with CP (*P* < .001) (Table 1). HH was observed in 10%, 26.7%, and 70.6% in MP <33%, MP 33%–39%, and MP> 40% groups, respectively.

**Table 1:** Comparison of clinical features according to the severity of migration percentage of hip displacement on pelvic radiography.

Variable	MP0 <0 33% (n0 =0 20)	MP 33–39% (n0 =0 15)	MP0 >0 40% (n0 =0 17)	*P* *
Age (years), (median, [interquartile range])	3.6 (2.4–5.7)	7.2 (3.3–10.9)	6.1 (4.6–12.6)	.015
Male, n (%)	12 (60.0)	7 (46.7)	8 (47.1)	.425
Nutritional status†				.360
Wasting	9 (47.4)	5 (33.3)	8 (47.1)	
Overweight & obesity	7 (36.8)	3 (20.0)	2 (11.8)	
No. of patients with hiatal hernia, n (%)	2 (10)	4 (26.7)	12 (70.6)	<.001

MP = migration percentage.

* *P* value < .05 was considered statistically significant.

† Weight for height was used for children aged < 2 years and the body mass index for children aged > 2 years.

### 3.2. Comparison of MP between children with and without HH

**Figure 2.**
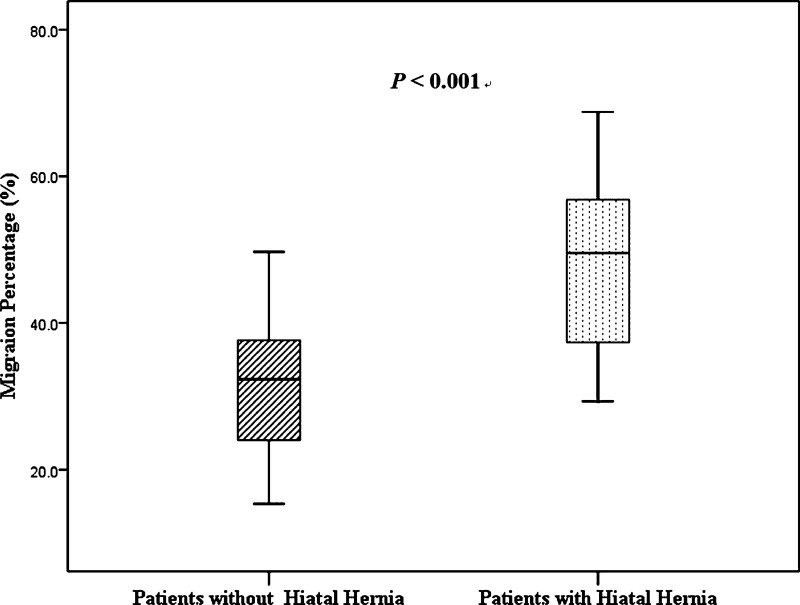
Migration percentage according to the presence or absence of hiatal hernia in children with cerebral palsy.

The median MP in pediatric CP patients without HH was 32.2% (range 26.4%–37.6%) and MP in children with CP with HH was 49.9% (36.0%–59.8%), revealing a significantly higher MP in those with HH (*P* < .001) (Fig. 2).

### 3.3. Optimal cutoffs and diagnostic accuracy of MP in detecting HH

**Figure 3.**
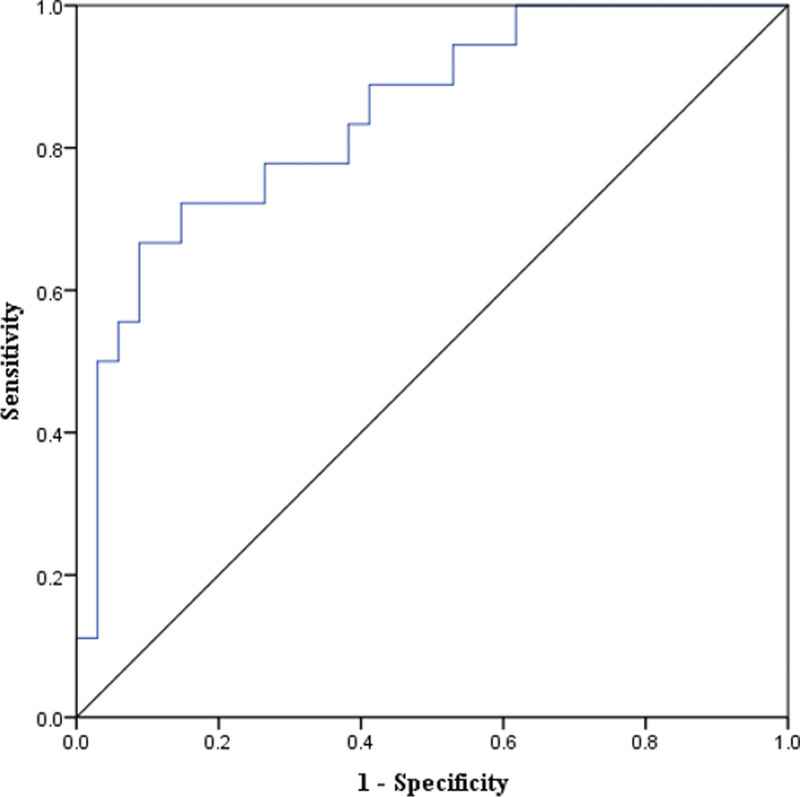
The ROC curve and its corresponding area under the curve of migration percentage to differentiate children with hiatal hernia from those without hiatal hernia, with an optimal cut-off of 36.5%. ROC = receiver operating characteristic.

The optimal MP cutoff of 36.5% distinguished the pediatric CP patients with HH from those without HH, with a sensitivity of 78%, specificity of 68%, positive predictive value of 56.0, and negative predictive value of 85.2%, respectively (Table 2). The area under the receiver operating characteristics curve of MP at an optimal cutoff of 36.5% was 0.845 (95% confidence interval, 0.734–0.956) (Fig. 3).

**Table 2:** Diagnostic accuracy of migration percentage in detecting the presence of hiatal hernia in children with cerebral palsy at the optimal cutoff of 36.5%.

	Children with HH	Children without HH	Total	Sensitivity (95% CI)	Specificity (95% CI)	PPV (95% CI)	NPV (95% CI)
Optimal cutoff of MP0 >0 36.5%							
Positive	14	11	25	77.8 (58.6–97.0)	67.6 (51.9–83.4)	56.0 (36.5–75.5)	85.2 (71.8–98.6)
Negative	4	23	27				
Total	18	34	52				

CI = confidence interval, HH = hiatal hernia, MP = migration percentage, NPV = negative predictive value, PPV = positive predictive value.

### 3.4. Comparison of presence of SSHH when no HH group is divided by MP cutoff of 36.5%

Of these 11 patients with an MP value greater than the cutoff values but no HH, 6 (54.5%) patients had SSHH by definition. In contrast, of the 23 patients whose MP values were less than the cutoff and had no HH, only 4 (17.4%) patients had SSHH.

## 4. Discussion

There are various causes of frequent occurrence of severe GERD and aspiration pneumonia in children with CP.^[30]^ And some of these causes are factors that cannot be corrected, and some of them are manageable. HH is one of the main causes of severe GERD in children with CP and has great clinical significance in that it can be treated.^[17]^ To date, despite the clinical importance of HH, the studies on pediatric patients have been lacking, mainly due to the diagnostic difficulties in approaching HH. Accordingly, if there is a non-invasive screening tool that can predict the high-risk group of HH, it may be of great help in assessing and managing the pediatric patients with CP in practice.

To our knowledge, this is the first study to reveal the correlation between the severity of hip displacement and HH in children with CP, and also the first study using simple radiography as a screening tool for detecting HH. In the present study, the results of MP were divided into three stages according to a general criterion for classifying the MP; MP < 33% is considered to be insignificant in hip dislocation, MP 33%–40% requires intensified observation, and MP > 40% requires surgical intervention.^[28,29]^ In this study, as the severity of MP increased, the proportion of HH increased in pediatric CP patients. In addition, a statistically significant difference was found when comparing the MP levels of patients without and with HH. The exact cause of a clear association between MP and incidence of HH is unknown, but our assumption is that this result is due to the fact that hip displacement and HH have common causative and aggravating factors. These results may support clinicians in screening high-risk patients for HH based on the MP, which can be easily obtained through a simple pelvic radiograph.

According to previous studies, hip displacement occurred as early as around 2 years of age, and it occurred more frequently with an increase in age because hip dislocation gradually progressed due to the complex action of several factors.^[22]^ In our study, it was confirmed that the patients with MP ≥ 30% were older than those with MP < 33%; these results were consistent with previous studies on hip displacement. Furthermore, as for HH, the patients with HH (median age 6.0 years, range 4.5–8.7 years) were also older than those without HH (median age 3.9 years, range 2.5–8.2 years); however, statistical significance was not demonstrated (*P* = .078).

The next step of our study was to determine the optimal cutoff values of MP to differentiate between the patients with HH from those without HH, which revealed that a cutoff MP of 36.5% was optimal with a sensitivity of 78% and a specificity of 68% with an area under the ROC curve of 0.845. Therefore, based on our study results, an MP beyond the suggested cutoff values on pelvic radiography can assist pediatricians to decide whether to perform further diagnostic investigations, such as EGD and UGIS to diagnose HH.

In our study, when 36.5% of MP was used as an optimal cutoff to detect HH in children with CP, 14 of 25 pediatric patients with an MP of 36.5% or higher were diagnosed with HH on further diagnostic investigations including EGD and UGIS, with a positive predictive value of 56.0%. Nevertheless, there were still 11 patients whose MP value exceeded the cutoff value of 36.5%, but did not have HH. In general, the proximal displacement of the GEJ that does not exceed 2 cm but exceeds 0.5 cm is defined as SSHH, and this case was not regarded as HH in our study. More research on SSHH is underway, and patients with SSHH tend to have more severe GERD compared to those without HH.^[31]^ Among patients without HH, the proportion of SSHH was much higher in patients with MP values higher than the cutoff values (54.5% vs 17.4%). These results can be interpreted as patients whose MP value exceeds the cutoff value have more clinically meaningful SSHH even if they do not have definite HH. This also indicates the association between the MP value and HH.

In recent years, hip surveillance programs have been increasingly implemented for patients with CP to diagnose hip dislocation early.^[22,32]^ Thus, all CP patients are regularly evaluated for hip dislocation by pelvic radiography according to the Gross Motor Function Classification System grade, and effective prevention and treatment are performed through early detection of hip displacement. By linking this program with the results of the present study, pediatric CP patients with high MP can undergo the evaluation for HH and GERD as well through consultation with pediatric gastroenterologists. This approach may be of great clinical significance to neurologically impaired patients by preventing severe GERD complications as early as possible.

Our study still has some limitations. Although a single investigator thoroughly reviewed all previous endoscopic images to evaluate HH, the retrospective design of the study could be a drawback. Another limitation of this study is a small number of patients despite being a 7-year retrospective study. This is probably because it is not clinically easy to perform invasive procedures such as EGD and UGIS in CP patients. It may also be a limitation that this study was conducted only on children with CP with symptoms. Therefore, it is still unclear whether the same results can be applied to patients with no clinical symptoms among children with CP with high MP. Based on the results of this study, it is necessary to implement further prospective studies without these limitations in the future. Nevertheless, our study has great clinical significance as this is the first study to screen high-risk groups of HH by simple radiography, with a relatively good diagnostic accuracy.

In conclusion, in children with CP, the severity of hip displacement was associated with the occurrence of HH. Identifying high-risk patients using MP, which can be easily confirmed by simple radiography, may be a useful and reliable tool to detect HH in children with CP at risk of developing HH.

## Acknowledgments

Not applicable.

## Author contributions

Conceptualization: In Hyuk Yoo, Hye Ran Yang.

Formal analysis: In Hyuk Yoo.

Investigation: In Hyuk Yoo.

Supervision: Hye Ran Yang.

Writing – original draft: In Hyuk Yoo.

Writing – review & editing: Hye Ran Yang.
